# Serological and histopathological investigation of brucellosis in cattle in Medea region, Northern Algeria

**DOI:** 10.14202/vetworld.2019.713-718

**Published:** 2019-05-28

**Authors:** El Aid Kaaboub, Nassim Ouchene, Nadjet Amina Ouchene-Khelifi, Djamel Khelef

**Affiliations:** 1 Institute of Veterinary Sciences, University of Saad Dahlab Blida1, 09000, Blida, Algeria; 2 National High Veterinary School of Algiers, 16000, Algeria

**Keywords:** Algeria, *Brucella*, cattle, histopathological analysis, seroprevalence

## Abstract

**Aim:**

This study was performed to determine the prevalence of bovine brucellosis in Medea region, Northern Algeria.

**Materials and Methods:**

The study was carried out on 495 non-vaccinated cattle, of which 280 (30 males and 250 females) belonged to 57 cattle farms and 215 cows were sampled at abattoirs of Medea. Sera collected from the cattle were tested using the Rose Bengal test and confirmed by histopathological analysis.

**Results:**

Serological examination revealed that 7/57 farms (12.28%) were infected, of which 7/280 (2.5%) cattle were seropositive. The prevalence in females and males was 2.4% (6/250) and 3.33% (1/30), respectively. No significant difference has been observed between females and males. Older animals (≥8 years) were infected more. The prevalence of infection was 9.1%. Seroprevalence of *Brucella* infection in cows that have already had abortion was higher compared with non-aborted cows (4.34% and 2.20%, respectively). In abattoirs, a total of 25 (11.62%) seropositive cows were detected, and the histopathological analysis was positive in all these cows.

**Conclusion:**

The study indicates that brucellosis indeed exists in cattle in Medea and shows that the meat of slaughtered cattle tested positive for brucellosis may constitute a real risk of transmission to both butchery personnel and consumers, which requires that the meat of infected animals should be analyzed before being marketed.

## Introduction

Brucellosis is one of the most important zoonotic diseases and considered as a major obstacle to livestock production in many developing countries worldwide. It is caused by bacteria of the genus *Brucella*, a Gram-negative facultative intracellular bacterium [1-5]. In cattle, brucellosis is caused by *Brucella abortus*, and it has a great economic incidence with reproductive failure characterized mainly by abortion during the last trimester of gestation, infertility and reduced milk production in females, and infertility, orchitis, and epididymitis in males [6-10]. Aborted fetuses and uterine secretions are the most important sources of infection. The transmission to the calves can be done vertically and through contaminated milk [11,12]. Artificial insemination with contaminated semen has been reported as a potential source of infection [13]. Brucellosis in humans caused habitually from an animal reservoir, and the majority of cases are attributed to *Brucella* *melitensis* [14,15]. Other *Brucella* species can rarely cause infection [16]. Human brucellosis is principally due to the consumption of contaminated milk, and other products originated from ruminants or by direct contact with infected animals, aborted fetuses, live vaccine strains, or by the manipulation of virulent *Brucella* species in the laboratory [17-19].

Clinical diagnosis in livestock is founded on the history of reproductive failures, and it must be confirmed by laboratory methods [20,21]. The serological tests are still frequently used in the diagnosis of brucellosis. Mostly based on serological evidence, brucellosis has been reported throughout much of Africa [22-25].

In Maghreb, the brucellosis epidemiological study remains poorly documented [23]. In Algeria, many programs for eradication of brucellosis in ruminants have been based on several strategies: Mass vaccination and/or testing and slaughter of infected animals [26]. However, the animal brucellosis in Algeria remains present. The pasteurization of dairy products is not systematic. Certain food habits (i.e., consumption of raw milk/cheese) and insufficient hygienic practices increase bacterial transmission to humans [27].

This study aimed to determine the prevalence of brucellosis in cattle using Rose Bengal test (RBT) and verify RBT results with the histopathological analysis of supramammary and retropharyngeal lymph nodes of the seropositive cattle slaughtered in abattoirs of Medea, Northern Algeria.

## Materials and Methods

### Ethical approval

Ethical approval is not necessary for such type of study. However, blood samples were collected as per standard collection procedure without any harm to animals.

### Study area

This study was performed in Medea region, Northern Algeria. Medea region is mountainous and is 630 m above sea level. It has a semi-arid climate characterized by hot summers, and cold and wet winters with a rainfall averaging 410 mm per year [28,29]. The study was conducted between September 2014 and May 2015 on a cattle farms and abattoirs of Medea region.

### Study population

The study was carried out on 495 non-vaccinated cattle consisted of 280 (30 males and 250 females) from 57 cattle farms and 215 cows from abattoirs of Medea. The information about each animal (age, sex, race, vaccination, and pregnancy) was recorded.

### Sample collection

Blood samples were collected from the jugular vein of each animal (in farms or abattoirs) using vacuum-dried tube. Each sample was identified using codes describing the specific animal and herd. Samples were centrifuged at 3000 g for 20 min, and the obtained serum was collected by a micropipette, placed in Eppendorf tubes, and tested by RBT.

In abattoirs, the retropharyngeal and supramammary lymph nodes from slaughtered cows were collected and conserved immediately in 10% formalin for histological analysis.

### Serological analyses of samples

RBT was used for the serology analysis. Sera samples were screened using RBT antigen according to Alton *et al*. [30]. Briefly, 30 µL of the test serum and 30 µL of RBT antigen were placed on the plate and then mixed carefully. The plate was agitated for 4 min and the degree of agglutination was recorded. Positives and negatives control sera were used for comparison.

The sample was considered positive if any agglutination was observed and negative if no agglutination was observed.

### Histopathological analysis of lymph nodes

Histological analysis of lymph nodes (retropharyngeal and supramammary) was performed for 25 slaughtered seropositive cows. Lymph nodes were fixed in 10% neutral buffered formalin for about 2 days. The specimens were processed by paraffin embedding method, sectioned 4–5 µm, and stained with hematoxylin and eosin according to Bancroft and Gamble [31] for histopathological examination. Lymph nodes of one seronegative cow were used as control lymph node.

### Statistical analysis

The statistical program used was R version 3.0.1 (R Core Team 2013, R Foundation for Statistical Computing, Vienna, Austria, URL: http://www.R-project.org). The Chi-square test was used for statistical analysis. Differences were considered statistically significant when p<0.05.

## Results

### Serological analysis

Serological examination revealed brucellosis infection of 7/57 farms (12.28%). A total of 7/280 (2.5%) serologically positive animals were detected in these farms. The prevalence in females and males was 2.4% (6/250) and 3.33% (1/30), respectively. No significant difference (p>0.05) has been observed between females and males (Table-1). According to the age of the animals, the prevalence of infection was significantly higher in animals over 8 years old (9.1%) (p<0.0001) (Table-1).

**Table-1:** Prevalence of *Brucella*-infected cattle in farms according to age and sex type.

Age and sex	No. examined animals	No. positive animals (%)	p-values
Age of animals (years)			
≤2	95	1 (1.05)	p<0.0001
2-5	130	1 (0.77)	
≥8	55	5 (9.1)	
Total	280	7 (2.5)	
Sex			
Males	30	1 (3.33)	p>0.05
Females	250	6 (2.4)	

Among the 280 cattle, the number of cows that have already had abortion was 23, of which one cow was seropositive, giving a prevalence of 4.34% (1/23). For non-aborted cows, the prevalence of 2.20% (5/227) was recorded. Statistical analysis revealed a significant difference (p<0.001). In abattoirs, a total of 25/215 (11.62%) serologically positive cows were detected in this study. Positive farms were located under quarantine by the veterinary authorities.

### Histopathological analysis of lymph nodes

**Figure-1:**
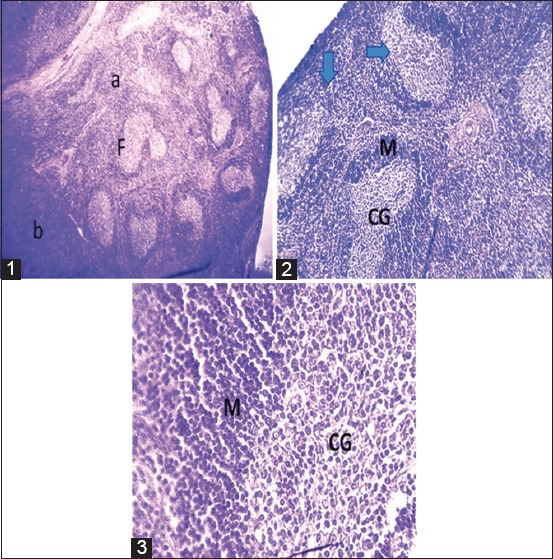
Control lymph node: (1) 40× g: (a) Cortical zone of a lymphatic ganglion with the presence of lymphoid follicle (F) with a central zone proliferative clear and dark peripheral zone. (b) Paracortical zone with a diffuse stroma containing lymphocytes not very visible at this magnification. The cortical and paracortical zone are remarkably isolated and individualized, showing clear histological limits. (2) 100× g: Details of the cortical zone with lymphoid follicles containing a clear germinal center (GC) surrounded by a dark peripheral zone (M) consisting of immature B lymphocytes. (3) 400× g: Details of the GC showing a lymphocyte concentration with a clear appearance delimited by a darker zone itself formed of immature lymphocyte cells. The appearance of the nucleus is indicative of the state of cellular activity.

The histopathological analysis was positive in all seropositive cows (n=25). In the control lymph node, the cortical and paracortical zone are remarkably isolated and individualized, showing clear histological limits. The lymphoid follicles are well visible in the cortical zone (Figure-1).

**Figure-2:**
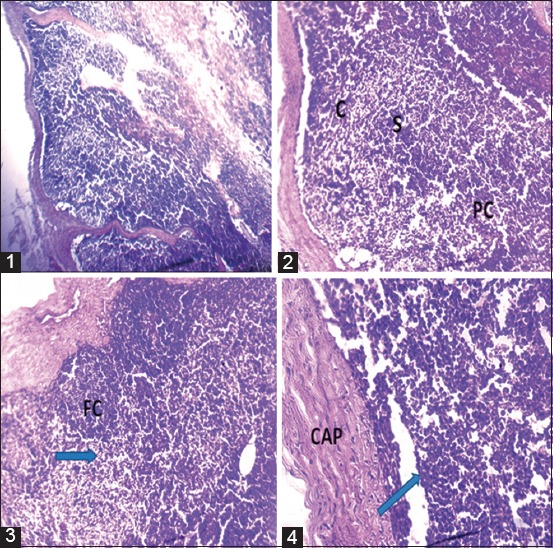
Acute form: Photomicrograph of a cattle lymph node showing: (1) 40× g: Disappearance of the lymphoid follicles in the cortical zone. (2) 100× g: Lymphoid hyperplasia, disappearance of the lymphoid follicle, and the corticoparacortical junction. (3) 100× g: Dehiscent cortical follicles, disappearance of corticoparacortical junction (arrow). (4) 400× g: Detail of the paracortical zone demonstrating lymphocytic hyperplasia with complete disappearance of the original histological structures.

In the acute brucellosis form, lymph node changes have been characterized as lymphocytic hyperplasia in the cortical and paracortical zone with disappearance of lymphoid follicles and corticoparacortical junction (Figure-2).

**Figure-3:**
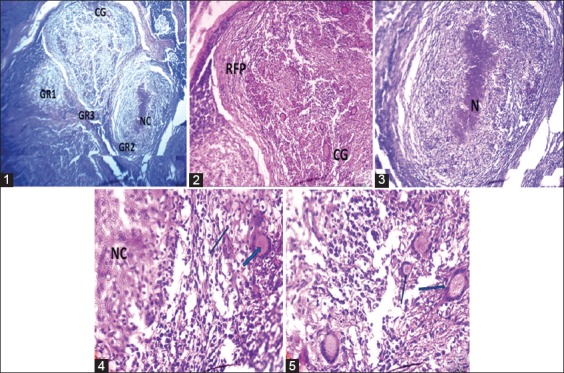
Chronic form: Photomicrograph of a cattle lymph node showing: (1) 40× g: Presence of the necrosis in the granuloma GR3 and giant cells clearly visible in GR2. (2) Details of Bang granuloma (100× g): Lymphocyte infiltration and fibroproliferative remodeling at the periphery of the granuloma. Presence of several giant cells. (3) Bang granuloma with necrotic center (N) (100× g). (4) Bang granuloma 400× g: Giant cell and epithelial cells within the inflammatory granuloma. (5) Bang granuloma (400× g): Several giant cells with eosinophilic cells within the Bang granuloma.

In the chronic brucellosis form, lymph node changes are characterized by granulomatous lymphadenitis with the presence of giant cells that are dispersed within a stroma invaded by hyperactivated epithelial cells or macrophages delimited by a massive infiltration of lymphocytes. Central necrosis was observed. Cortical and paracortical structures have disappeared, giving way to tissue mainly consisting of easily identifiable lymphocytes and other types of inflammatory cells that are difficult to identify, often requiring identification by immunohistochemistry. This typical inflammatory entity of brucellosis is described as Bang granuloma (Figure-3).

## Discussion

In this study, we revealed the seroprevalence of *Brucella* infection in cattle in Medea region, and for the best of this study, for the 1^st^ time in Algeria; seropositive cases were confirmed by histological analysis.

In Algeria, animal brucellosis was diagnosed for the 1^st^ time in 1907 in goats [32]. After the Algeria independence in 1962, the first brucellosis study in cattle in 1969 showed high prevalence (23%) [33] compared to neighboring Tunisia (1.94%) and Morocco (1.4%) [34]. There was a regression in seroprevalence of 5% in 1990, possibly as a result of health and sanitary measures [35].

In 1995, the Algerian Veterinary Services initiated a multiannual national program to control ruminants’ brucellosis based on sanitary prevention with the screening-sloughing operations [36]. Through this program, statistical evaluation revealed amelioration of the sanitary statute regarding animal brucellosis with prevalences ranging from 5% in 1990 to 0.76% in 2014. However, this program evaluated only 6% of the Algerian cattle population [36].

In our study, the serological evidence of brucellosis indicated that 7 of 57 herds were infected accounting for 12.28% herd seroprevalence which is in concordance with Kardjadj [37], who reported a seroprevalence of 12% in Algeria. This means a significant reduction in the cattle brucellosis seroprevalence compared to that stated previously by Aggad and Boukraa [38] who reported a seroprevalence of 26.3%, suggesting an improvement of the brucellosis sanitary status in Algerian cattle.

Within-herd brucellosis seroprevalence revealed in our study was 2.5% (7/280) in agreement with Khaldi *et al*. [39] and Rechidi-Sidhoum *et al*. [40], where they reported 1.1% and 0.97% of individual seroprevalence, respectively, in Northwest Algeria. Higher seroprevalences of 33.33%, 10.4%, and 8.2% were reported by Kardjadj [37], Bouzid *et al*. [41], and Aggad and Boukraa [38], respectively, in Algeria. Abdelhadi *et al*. [42] revealed a prevalence of 6.52% using ELISA test in West Algeria.

In our study, older animals (≥8 years) are the most infected (p<0.0001). Many authors have reported that *Brucella* infection is less common in young cattle than in adults [43-45]. The higher prevalence in older cattle can be attributed to constant exposure of the cattle overtime to the infectious agent [46].

In our study, the prevalence of brucellosis between sexes was not different significantly. This is consistent with reports by Bayemi *et al*. [47] and Kubuafor *et al*. [48]. Regardless of its acknowledgment as an important economic and public health problem and the availability of proven control means and the application of the screening-sloughing operations since 1995, brucellosis continues to occur in Algerian cattle herds causing severe economic losses [49]. A large number of unpublished studies in Algeria had suggested an association between *Brucella* seropositivity and abortion in cattle [37]. Indeed, our results confirm that seroprevalence is higher in cattle with abortion history when compared with cattle with non-abortion history (4.34% and 2.20%, respectively) (p<0.001).

In Algeria, the serological test used in bovine brucellosis control is RBT, which seems to be acceptable. However, a confirmation test is required [38]. In our study, the histopathological analysis of lymph nodes of seropositive slaughtered cows was used to confirm the RBT results.

RBT is a very sensitive and quick test, inexpensive, and easy to perform. False-negative reactions are rare [50]. In our study, the histopathological analysis was positive for all seropositive cows, which indicates that RBT is a very specific test for the diagnosis of bovine brucellosis and the false-positive reactions almost absent.

After infection, *Brucella* localizes in various lymph nodes of female cattle such as supramammary, retropharyngeal, and mandibular lymph nodes; internal and external iliac lymph nodes and uterus [51]. Supramammary lymph node is the most common site for *Brucella* localization [52]. The most observed lesion involved the lymph nodes, which were remarkably hypertrophic and had follicular hyperplasia with a few giant cells and macrophages [51]. In our study, the histopathological analysis was performed for retropharyngeal and supramammary lymph nodes, and the observed lesions clearly indicate a *Brucella* infection.

## Conclusion

This study revealed overall moderate seroprevalence at individual cattle level and high seroprevalence at herd level. The meat of slaughtered cattle tested positive for brucellosis may be constituted a real risk of transmission to both butchery personnel and consumers, which requires that the meat of infected animals should be analyzed before being marketed. It is, therefore, important that farmers seek veterinary advice on the brucellosis status of their animals, particularly those used for breeding purposes. The veterinary authorities in Algeria must make more efforts to fight to improve their surveillance systems and to disseminate information to other relevant stakeholders in order to eradicate this important zoonosis.

## Authors’ Contributions

EK collected and processed the samples. NO, NAO and DK analyzed and interpreted the result. NO drafted the manuscript. All authors participated in the draft and revision of the manuscript. All authors read and approved the final manuscript.
